# Clinical utility of the cogstate brief battery in identifying cognitive impairment in mild cognitive impairment and Alzheimer’s disease

**DOI:** 10.1186/2050-7283-1-30

**Published:** 2013-12-23

**Authors:** Paul Maruff, Yen Ying Lim, David Darby, Kathryn A Ellis, Robert H Pietrzak, Peter J Snyder, Ashley I Bush, Cassandra Szoeke, Adrian Schembri, David Ames, Colin L Masters, for the AIBL Research Group

**Affiliations:** The Florey Institute of Neuroscience and Mental Health, University of Melbourne, Parkville, Victoria Australia; CogState Ltd, Melbourne, Victoria Australia; Academic Unit for Psychiatry of Old Age, Department of Psychiatry, The University of Melbourne, Kew, Victoria Australia; National Ageing Research Institute, Parkville, Victoria Australia; Department of Psychiatry, Yale University School of Medicine, New Haven, CT USA; Lifespan Hospital System & Department of Neurology, Warren Alpert Medical School of Brown University, Providence, RI USA; CSIRO Preventative Health Flagship, Parkville, Victoria Australia

## Abstract

**Background:**

Previous studies have demonstrated the utility and sensitivity of the CogState Brief Battery (CBB) in detecting cognitive impairment in Alzheimer’s disease (AD) and mild cognitive impairment (MCI) and in assessing cognitive changes in the preclinical stages of AD. Thus, the CBB may be a useful screening tool to assist in the management of cognitive function in clinical settings. In this study, we aimed to determine the utility of the CBB in identifying the nature and magnitude of cognitive impairments in MCI and AD.

**Methods:**

Healthy adults (n = 653) adults with amnestic MCI (n = 107), and adults with AD (n = 44) who completed the CBB participated in this study. Composite Psychomotor/Attention and Learning/Working Memory scores were computed from the individual CBB tests. Differences in composite scores were then examined between the three groups; and sensitivity and specificity analyses were conducted to determine cut scores for the composite scores that were optimal in identifying MCI- and AD-related cognitive impairment.

**Results:**

Large magnitude impairments in MCI (g = 2.2) and AD (g = 3.3) were identified for the learning/working memory composite, and smaller impairments were observed for the attention/psychomotor composite (g’s = 0.5 and 1, respectively). The cut-score associated with optimal sensitivity and specificity in identifying MCI-related cognitive impairment on the learning/working memory composite was -1SD, and in the AD group, this optimal value was -1.7SD. Both composite scores showed high test-retest reliability (*r* = 0.95) over four months. Poorer performance on the memory composite was also associated with worse performance on the Mini Mental State Exam and increasing severity on the Clinical Dementia Rating Scale sum of boxes score.

**Conclusions:**

Results of this study suggest that the CogState learning/working memory composite score is reduced significantly in CI and AD, correlate well with measures of disease classification and are useful in identifying memory impairment related to MCI- and AD.

## Background

The importance of screening for dementia in individuals at risk of neurodegenerative diseases is now widely accepted (Snyder 2013). While advances in neuroimaging and fluid biomarkers show much promise for identifying early Alzheimer’s disease (AD), neuropsychological testing remains the cornerstone of early disease recognition (Albert et al. 2011; McKhann et al. 2011). Unfortunately, most neuropsychological test batteries shown to be sensitive to early AD require substantial time and expertise for both administration and scoring and this can limit their potential for use in wide-scale screening (Fredrickson et al. 2010). While some brief bedside cognitive screening instruments (i.e. measures that require less than 30 minutes for administration) such as the Mini Mental State Examination (MMSE) (Folstein et al. 1975) and Montreal Cognitive Assessment (MoCA) (Nasreddine et al. 2005) have been shown to be useful in case finding studies of AD and MCI, their relative lack of sensitivity to detecting subtle cognitive impairment has been well documented (McKhann et al. 2011; Proust-Lima et al. 2007) as has their potential for idiosyncratic errors in administration (Miller et al. 2008; Miller et al. 2011). Furthermore, although items on these bedside screening instruments are selected to assess a wide variety of cognitive domains, subscale scores on these instruments generally have low validity and reliability (Strauss et al. 2006).

The CogState Brief Battery (CBB) is a brief, computer-administered cognitive test battery that requires approximately 10 minutes for administration and consists of four cognitive tasks that measure psychomotor function, attention, working memory and memory (Darby et al. 2012; Fredrickson et al. 2010; Maruff et al. 2009). The sensitivity of the CBB to detect cognitive impairment in several neurodegenerative conditions has been demonstrated in prior work (Darby et al. 2009; Hammers et al. 2012; Lim et al. 2012a). Given that the CBB is computerized, the administration, scoring and reporting is automated and highly standardized. Each task in the battery is constructed using playing cards as stimuli with the test taker required to answer only “yes” or “no” on each trial in accord with a simple rule. The simple stimuli, rules and responses have been combined to generate cognitive paradigms that have been well-validated in neuropsychological and cognitive studies. These include measures of psychomotor function (Detection task), visual attention (Identification task), working memory (One Back task) and visual learning set within a pattern separation model (One Card Learning task, (Fredrickson et al. 2010; Maruff et al. 2009)). The simplicity of the CBB has allowed it to be applied successfully to the measurement of cognitive function in healthy older adults and in adults with clinically diagnosed and prodromal AD (Darby et al. 2009; Lim et al. 2012a, b). These studies have found that performance on the CBB working memory and learning tasks are sensitive to cognitive impairment in clinically diagnosed AD as well as its prodromal stage; amnestic MCI. Furthermore, the CBB was designed specifically for repeated administration, as it can be administered repeatedly without generating significant practice effects (Collie et al. 2003; Falleti et al. 2006), including in healthy older people (Fredrickson et al. 2010). The CBB has been shown to be sensitive to AD-related cognitive decline in healthy older adults and in adults with amnestic MCI (Darby et al. 2002,2012; Lim et al. 2013a, b) as well as to improvement in cognition arising from treatment with putative cognitive enhancing drugs such as donepezil (Jaeger et al. 2011), histamine H3 antagonists (Nathan et al. 2013) and testosterone (Davison et al. 2011) in older people.

Recent data from studies using the CBB suggests that composite scores, which are constructed from aggregating performance on the Detection and Identification tasks (i.e., an attention/psychomotor composite) and the learning and working memory tasks (i.e., a learning/working memory composite) may have greater sensitivity to both AD-related cognitive impairment and decline when compared to scores from the individual CBB tasks (Lim et al. in press 2012b, c). This increased sensitivity of cognitive composite scores over individual test scores is consistent with current neuropsychological models that emphasise the benefit of composite scores in clinical research (Nuechterlein et al. 2008).

While the CBB is not intended to replace formal neuropsychological assessment, the results of these recent studies do converge to suggest that it may be useful as a screening test for AD-related cognitive impairment in clinical settings. However, the clinical utility of the CBB in screening for AD-related cognitive impairment has not been established formally. To achieve this, it is necessary to compute estimates of sensitivity and specificity of each composite score and identify their optimal value for the identification of cognitive impairment related to both AD and MCI. It is also necessary to understand the nature of any relationship between each composite measure and cognitive impairment across disease severity. Finally, establishing the reliability and stability of these composite scores would facilitate the use of composite cognitive measures to monitor changes in cognitive function in clinical or prodromal AD.

The main aim of this study was to determine the sensitivity, specificity and reliability of the CBB composite scores for the detection and monitoring of cognitive impairment in aging and dementia (Lim et al. 2012a, b). The first hypothesis was that the attention/psychomotor and learning/working memory composites would be sensitive to AD-related cognitive impairment although the sensitivity of the learning/working memory composite would be greater than that of the attention/psychomotor composite. We then examined the relationship between each cognitive composite score and disease severity across the clinical groups. Our second hypothesis was that on reassessment, both cognitive composite scores would show high test-retest reliability and stability in healthy adults, amnestic MCI and AD.

## Methods

### Participants

Participants in the current study were recruited from the Australian Imaging, Biomarkers and Lifestyle (AIBL) Study of Ageing (Ellis et al. 2009; Rowe et al. 2010) and from hospital clinics specializing the diagnoses of AD who had completed the CBB successfully as part of their assessment (Lim et al. 2012a). The process of recruitment and diagnostic classification been described in detail previously for the AIBL (Ellis et al. 2009) and clinical samples (Maruff et al. 2004). Of the AIBL participants who had completed the CBB, 659 healthy adults (HA), 72 adults who met clinical criteria for amnestic MCI and 51 adults who met clinical criteria for mild to moderate AD (Ellis et al. 2009) were recruited into the study. For the hospital clinical sample 35 patients who met clinical criteria for amnestic MCI were recruited (Maruff et al. 2004). Briefly, all patients underwent a detailed diagnostic workup by clinician specializing in AD on the basis of clinical, neuropsychological and structural neuroimaging data. All cases of amnestic MCI were classified using established criteria (Petersen et al. 1999; Winblad et al. 2004). All cases of AD met NINCDS-ADRDA criteria for AD (McKhann et al. 1984). To increase the reliability of classification, all individuals classified with MCI and AD were required to meet the criteria for these clinical classifications on two consecutive assessments. Data from the CBB was not used by clinicians to classify any individual’s clinical status. For participants with AD, additional inclusion criteria included a score of 18 to 26 on the MMSE (Folstein et al. 1975). The severity of dementia was rated in patients with AD and MCI using the Clinical Dementia Rating (CDR) scale to provide a sum of boxes score and an overall CDR score (Morris 1983). For all participants, exclusion criteria for the study included: schizophrenia; depression (15-item Geriatric Depression Score (GDS) of 6 or greater); Parkinson’s disease; cancer (except basal cell skin carcinoma) within the last two years; symptomatic stroke; uncontrolled diabetes; or current regular alcohol use exceeding two standard drinks per day for women or four per day for men. None of the control or MCI group were taking psychotropic drugs or cholinesterase inhibitors although each of the patients with AD were taking cholinesterase inhibitors. Demographic and clinical characteristics of the HC, MCI and AD groups are shown in Table 1. The study complied with the regulations of three institutional research and ethics committees (Ellis et al. 2009), and all participants gave written informed consent prior to participation in the study. To assess test-retest reliability, we re-assessed 115 HA, 47 adults with MCI, and 43 adults with AD who underwent serial assessments on the computerized cognitive battery. These individuals were assessed monthly over four months (Lim et al. 2013b). The process of recruitment and additional inclusion and exclusion criteria for this subgroup of AIBL participants has been described in detail previously (Lim et al. 2013b).

**Table 1:** **Demographic and clinical characteristics for each clinical group**

	HC (***n*** = 659)	MCI (***n*** = 107)	AD (***n*** = 51)
	Mean (SD)	Mean (SD)	Mean (SD)
Percentage females^+^	57.8%	50.5%	51.0%
Age (years)	69.5 (6.6)	75.7 (7.5)	79.3 (7.2)
MMSE	28.7 (1.4)	26.1 (2.1)	19.8 (3.8)
CDR-SB	0.06 (0.3)	1.39 (1.2)	5.87 (2.4)
Premorbid IQ	108.35 (7.3)	105.9 (9.0)	103.2 (8.4)
Education level ^med^	12 (9–15)	12 (9–15)	12 (9–15)
HADS depression	2.6 (2.2)	3.3 (2.4)	3.8 (3.1)
HADS anxiety	4.3 (2.9)	4.1 (2.6)	4.7 (3.7)
Detection speed*	100.0 (10.0)	94.26 (13.7)	91.72 (13.5)
Identification speed*	100.0 (10.0)	87.62 (16.4)	84.12 (15.4)
One card learning accuracy*	100.0 (10.0)	83.74 (11.6)	78.42 (15.1)
One back accuracy*	100.0 (10.0)	79.18 (13.1)	70.14 (16.3)

Note: + = percentage of clinical group, med = median (range), * = mean score =100 and SD score = 10 because the mean and SD of the controls was used to standardize the data for each individuals performance on each cognitive task. One way ANOVAs indicated significant differences between groups on age, premorbid IQ, and depressive symptoms, all *p*’s < 0.001. MMSE = Mini Mental State Examination; CDR-SB = Clinical Dementia Rating Scale, Sum of Boxes Score; HADS = Hospital Anxiety and Depression Scale.

### Measures

#### Demographic and clinical characteristics

Participants underwent a series of comprehensive demographic, health and cognitive tests performed by trained research assistants under the supervision of licensed clinical neuropsychologists. Participants’ age was based on self-report, and this information was corroborated by a family member. Additionally, the MMSE, CDR, Wechsler Test of Adult Reading (WTAR) (Wechsler 2001) and the Hospital Anxiety and Depression Scale (HADS) (Snaith & Zigmond 1986) were administered to participants to measure overall cognitive impairment, general clinical function, premorbid IQ, and level of anxiety and depressive symptoms, respectively.

#### CogState brief battery

The four tasks from the CBB have been described in detail previously (Darby et al. 2012; Lim et al. 2012a, b), and they are summarized here. On each trial of each task, a single playing card stimulus was presented in the centre of the computer screen. The values, color and suit of the playing cards were determined by the requirements of each task. At the presentation of each playing card stimulus, participants were required to respond either “yes” or “no” by pressing a “yes” or “no” button attached to the computer through a USB port. The yes button was always placed on the right and pressed with the right hand and the no button was placed on the left and pressed with the left hand. Patients were instructed to press the “yes” or “no” button as quickly and as accurately as possible. At the beginning of each task, task rules were presented on the computer screen, and also given verbally to the participant by the supervisor. This was followed by an interactive demonstration in which participants practiced the task. Once the practice trials were complete, the task began. The four tasks were presented in the same order. For each task, the speed and accuracy of each response to each trial was recorded and expressed as a mean reaction time (in milliseconds) and accuracy (proportion correct). For each task a single performance measure has been selected on the basis that it comes from a normal data distribution, has no floor or ceiling effects, does not have restricted range and has good reliability, stability and sensitivity to change (Fredrickson et al. 2010; Hammers et al. 2011). The tasks from the CBB are described in their order of administration below.

The Detection (DET) task is a simple reaction time test shown to measure psychomotor function. In this task, the participant must attend to the card in the center of the screen and respond to the question “has the card turned over?” Participants were instructed to press the “Yes” button as soon as the card turns face up. The face of the card is always the same generic joker card. The task ends after 35 correct trials have been recorded. Trials on which anticipatory responses occurred were excluded and another trial was given so that all participants completed the 35 trials. The primary performance measure for this task was reaction time in milliseconds (speed), which was normalized using a logarithmic base 10 (log_10_) transformation.

The Identification (IDN) task is a choice reaction time test shown to measure visual attention. In this task, the participant must attend to the card in the center of the screen, and respond to the question “Is the card red?” Participants were required to press the “Yes” button if it is and the “No” button it is not. The face of the cards displayed were either red or black joker cards in equivalent numbers in random order. These cards were different to the generic joker card used in the DET task. The task ends after 30 correct trials. Trials on which anticipatory responses occurred were excluded and another trial was given so that all participants completed the 30 trials. The primary performance measure for this task was reaction time in milliseconds (speed), which was normalized using a log_10_ transformation.

The One Card Learning (OCL) task is a continuous visual recognition learning task that assesses visual learning within a pattern separation model (Yassa et al. 2010). Theoretical models of pattern separation model specify that information is organized in orthogonal and distinct non-overlapping representations so that that new memories can be stored rapidly without interference (Norman & O'Reilly 2003). In this task the participant must attend to the card in the center of the screen and respond to the question “have you seen this card before in this task?” If the answer was yes, participants were instructed to press the “Yes” button, and the “No” button if the answer was no. Normal playing cards were displayed (without joker cards). In this task, six cards are drawn at random from the deck and are repeated throughout the task. These four cards are interspersed with distractors (non-repeating cards). The task ends after 80 trials, without rescheduling for post-anticipatory correct trials. The primary performance measure for this task was the proportion of correct answers (accuracy), which was normalized using an arcsine square-root transformation.

The One-Back (OBK) task is a task of working memory and attention. Similar in presentation to the OCL task, participants must attend to the card in the center of the screen and respond to the question “is this card the same as that on the immediately previous trial?” If the answer was yes, participants were instructed to press the “Yes” button, and the “No” button if the answer was no. The task ends after 30 correct trials. A correct but post-anticipatory response led to scheduling of an extra trial. The primary performance measure for this task was the proportion of correct answers (accuracy), which was normalized using an arcsine square-root transformation.

### Data analysis

For each participant, each performance measure from the four tasks in the CBB was computed as reported previously (Lim et al. 2012a). For each performance measure, the mean and standard deviation (SD) was computed for the HA group according to their age in deciles (e.g., 51–60, 61–70, 71–80, 81–90). These means and SDs were then used to standardize scores on each of the four cognitive tasks for each participant. A learning/working memory composite score was computed by averaging the standardized scores for the OCL and OBK tasks, and an attention/psychomotor function composite score was computed by averaging the standardized scores for the DET and IDN tasks. For each individual, both composite scores were then re-standardized using the mean and SD for each composite score computed from the HC group and then transformed once more so that each had a mean of 100 and a standard deviation of 10. This was achieved by first multiplying each standardized score by 10 and then adding 100. If data for one or both of the tasks that contributed to each composite was missing, the composite score was not computed. There was no missing data for the attention/psychomotor function composite and 26 (HA = 17 cases, AD = 9 cases) missing data for the learning/working memory composite score.

To evaluate the first hypothesis that the composite scores would be sensitive to AD-related cognitive impairment, we conducted two analysis of covariance (ANCOVA), with age, premorbid IQ, and level of depressive symptoms entered as covariates. For each composite score, Hedge’s *g* was used to quantify the magnitude of impairment in each of the clinical groups relative to the healthy controls. We also determined the extent to which performance on each composite was worse in the AD group than in the MCI using ANCOVA with age, premorbid IQ, and level of depressive symptoms entered as covariates. Once again for each comparison Hedge’s *g* was used to quantify the magnitude of impairment in the AD group relative to the MCI group. Receiver operating characteristic (ROC) curves were then generated to illustrate the relationship between clinical sensitivity and specificity of each composite for classification of MCI and AD groups, as measured by the area under the curve (AUC) statistic. AUC values were compared to those obtained for the MMSE in the same analyses with statistical significance indicated when 95% confidence intervals for each estimate did not overlap. For classification of cognitive impairment in MCI and AD, the value of each composite score that provided the optimal balance between sensitivity and specificity was identified from the ROC curve using Youden’s J statistic (Swets 1996). The predictive power of the combination of the optimum cut-score for each composite in predicting MCI and AD was then determined by computing the odds ratios for the classification of cognitive impairment in each clinical group (versus the HC group). Finally the relationship between the cognitive composite scores and disease severity was determined by collapsing data for the MCI and AD group and classifying each individual according to their score on the CDR Sum of Boxes score. Curve fitting analysis was then used to determine the extent to which scores on each of the cognitive composites was associated with increased CDR Sum of Boxes scores.

To evaluate our second hypothesis that the cognitive composite scores would show high test-retest reliability and stability, we computed mean change scores and test-retest reliability statistics over four months for the two CogState composite scores. This was conducted in a subgroup of AIBL participants who had consented to serial computerized cognitive assessments (Lim et al. 2013b). Average measure intraclass correlation coefficients (ICC) were used to compute the test-retest reliability of the two composites, in both the total group and in each clinical classification group separately.

## Results

### Cognitive function in healthy controls

In the HA group, the attention/psychomotor composite was not associated significantly with premorbid IQ (*r* = 0.07, *p* >0.05) or level of education. It was associated significantly with levels of depressive (*r* = 0.11, *p* < 0.05) and anxiety symptoms (*r* = 0.10, *p* < 0.05). The learning/working memory composite was not associated significantly with premorbid IQ (*r* = -0.06, p > 0.05), or levels of depressive (*r* = 0.02, p > 0.05), or anxiety symptoms (*r* = 0.01, p > 0.05).

### Magnitude of cognitive impairment in MCI and AD

As has been reported previously (Lim et al. 2012a), comparison of the demographic variables between clinical groups indicated significant differences in age, premorbid IQ, and level of depressive symptoms (see Table 1). As such, these variables were included as covariates in comparisons of the CBB composite measures between groups.

Results of the ANCOVAs revealed statistically significant group differences for the learning/working memory composite, *F*(2,769) = 305.56, *p* < 0.001, and the attention/psychomotor function composite, *F*(2,794) = 26.52, *p* < 0.001. Post-hoc comparisons indicated that adults with MCI and AD performed significantly worse than HC on the learning/working memory composite, and the magnitudes of these differences were, by convention, large (MCI *g* = 2.15, 95% CI = 1.91, 2.38; AD *g* = 3.18, 95% CI = 2.91, 3.28). The AD group also performed significantly worse than the MCI group on the learning/working memory score with this difference moderate in magnitude (*g* = 0.84 95% CI = 0.49, 1.18; p < 0.01). Adults with MCI and AD also performed significantly worse than HA on the attention/psychomotor composite, although these differences were moderate-to-large in magnitude (MCI *g* = 0.51, 95% CI = 0.30, 0.72; AD *g* = 1.03, 95% CI = 0.73, 1.33). The AD group also performed significantly worse than the MCI group on the attention/psychomotor function score with the differences moderate in magnitude (is *g* = 0.40 95% CI = 0.07, 0.74).

### Sensitivity and specificity of CBB composite scores in assessing cognitive impairment in MCI and AD

**Figure 1:**
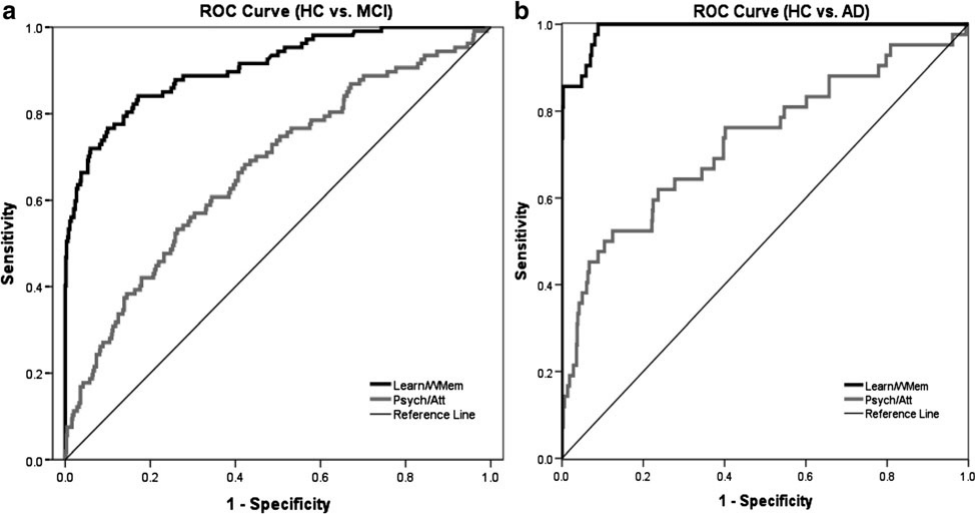
**ROC curve for performance of the MCI group (a) and the AD group (b) relative to the HC group on the learning/working memory composite and the attention/psychomotor composite.**

Inspection of the AUC statistics from the ROC analyses indicated that, by convention, the ROC curves for the learning/working memory composite showed excellent classification accuracy in both MCI and AD ((Swets 1996); Table 2; Figure 1). Accuracy of classification of both MCI and AD was lower for the attention/psychomotor composite (see Table 2, Figure 1). AUC values for the learning/working memory composite were significantly larger (i.e. no overlap between 95% CIs for AUC values) than for those for the attention/psychomotor composite and for classifying cognitive impairment in both MCI and AD (Table 2). Using the same criteria, the AUC for the learning/working memory composite was also significantly greater than the AUC for MMSE for classifying cognitive impairment in MCI (Table 2). Inspection of the Youden J statistics for the ROC curve for the learning/working memory composite indicated that the cut score that had optimal sensitivity and specificity in classifying cognitive impairment in MCI was 90 (i.e., z < = -1 SD). Application of this same cut score to classification of cognitive impairment in AD yielded a sensitivity of 100% at the same specificity (Table 2).

**Table 2:** **Areas under ROC curves for MCI and AD groups relative to healthy controls**

Composite	Clinical group	Sensitivity (95% CI) score < 90	Specificity (95% CI) score < 90	Area under ROC curve (95% CIs)	Standard error	***p***
Psychomotor/attention	AD	52.9% (38.5%, 67.1%)	85.7% (82.8%, 88.3%)	0.73 (0.64, 0.82)	0.05	< 0.0001
	MCI	41.1% (31.7%, 51.1%)	85.7% (82.8%, 88.3%)	0.67 (0.61, 0.73)	0.03	< 0.0001
		Score < 90	Score < 90			
Learning/working memory	AD	100.0% (91.5%, 100.0%)	84.7% (81.7%, 87.4%)	0.99 (0.98, 1.00)	0.01	< 0.0001
	MCI	80.4% (71.6%, 87.4%)	84.7% (81.7%, 87.4%)	0.91 (0.87, 0.94)	0.02	< 0.0001

Note: ROC = receiver operating characteristic; MCI = mild cognitive impairment; AD = Alzheimer’s disease; Attention/psychomotor composite = average of the standardized Detection and Identification scores; Learning/working memory composite = average of the standardized One Card Learning and One Back scores; MMSE = Mini Mental State Examination.

### Prediction of MCI and AD from combined composite scores

Table 3 shows the odds ratios for classification of MCI or AD (versus HA) for the combination of cognitive impairment of a score ≤90 on the learning/working memory composite and >/=90 on the attention/psychomotor composite. This analysis showed that with these cut scores, individuals were 26 times more likely to meet clinical criteria for MCI, and 30 times more likely to meet clinical criteria for AD.

**Table 3:** **Odds ratio, with impaired memory defined as scores of < 90**

	Normal memory normal attentional function (N)	Impaired memory normal attentional function (N)		Odds ratio (accuracy impaired)	***p***
Healthy controls (HC)	480	84			
Mild cognitive impairment (MCI)	12	55	HC vs. MCI	26.19 (13.45, 50.98)	< 0.0001
Alzheimer’s disease (AD)	4	21	HC vs. AD	30.00 (10.05, 89.60)	<0.0001

### Relationship to disease severity

For the relationship between MMSE scores and the attention/psychomotor composite, trend analysis indicated no statistically significant relationships in any clinical group. The relationship between MMSE scores and the learning/working memory composite was best described by a linear function in both the MCI (*r* = 0.38) and AD (*r* = 0.12) groups, although this relationship was statistically significant only for the MCI group.

**Figure 2:**
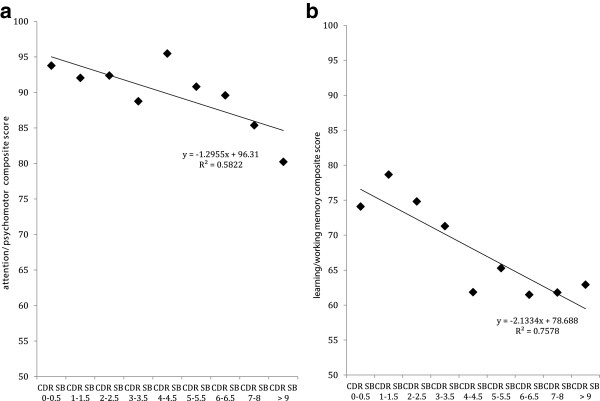
**Relationship between performance on the CDR Sum of Boxes and the attention/psychomotor composite (a) and the learning/working memory composite (b) in individuals with MCI and AD.** The diamond markers on each figure represent the mean composite score for each group of individuals with the same score on the CDR-SOB.

For the relationship between CDR sum of boxes scores and the attention/psychomotor composite, trend analysis indicated that when both MCI and AD groups were collapsed, there was a statistically significant linear relationship between increasing disease severity and worse performance on the attention/psychomotor composite (Figure 2a). Similarly, statistically significant linear relationships were observed between CDR sum of boxes scores and the learning/working memory composite when both the MCI and AD groups were collapsed (Figure 2b).

### Test-retest reliability

The ICC for both composites are shown in Table 4. When considered according to clinical classification, both composites demonstrated high (i.e., *r* > 0.70) test-retest reliability over a four month assessment period and these estimates were equivalent between the clinical groups (see Table 4).

**Table 4:** **Test-retest reliability and group mean (standard deviation) of each clinical group over a four month assessment period**

				Month 1		Month 2		Month 3		Month 4	
	Composite	ICC (95% CI)	***p***	Mean	SD	Mean	SD	Mean	SD	Mean	SD
Overall	Attention	0.90 (0.87, 0.92)	<.0001	96.46	11.97	95.79	15.65	95.66	11.79	97.37	23.16
	Memory	0.95 (0.93, 0.96)	<.0001	93.03	14.09	94.16	13.3	95.17	14.6	96.39	14.76
HC	Attention	0.94 (0.92, 0.96)	<.0001	100	8.86	98.86	9.08	99.26	8.81	101.27	27.59
	Memory	0.78 (0.70, 0.85)	<.0001	99.92	8.05	100.73	8.37	102.61	8.86	104.57	7.39
MCI	Attention	0.94 (0.90, 0.97)	<.0001	95.69	11.73	93.66	11.6	93.9	12.53	95.42	11.33
	Memory	0.86 (0.78, 0.92)	<.0001	92.18	10.99	92.16	9.51	92.53	11.03	93.89	10.02
AD	Attention	0.77 (0.58, 0.89)	<.0001	86.76	14.83	88.96	28.53	86.56	13.8	88.04	14.65
	Memory	0.91 (0.84, 0.96)	<.0001	73.4	13.07	75.95	12.26	75.1	13.11	75.16	13.46

Note: ICC = Intra-class correlation coefficient; HC = healthy controls; MCI = mild cognitive impairment; AD = Alzheimer’s disease; Attention/psychomotor composite = average of the standardized Detection and Identification scores; Learning/working memory composite = average of the standardized One Card Learning and One Back scores.

## Discussion

Results of this study supported our first hypothesis that the learning/working memory composite and the attention/psychomotor composite, derived from the outcome measures on the CBB, would be sensitive to detecting cognitive impairment in MCI and AD. In AD, we observed a large impairment for both cognitive composite scores, although the magnitude of impairment on the learning/working memory composite was much greater than that for the attention/psychomotor composite. Neuropsychological models of the cognitive tasks that contribute to the learning/working memory composite suggest that normal performance on these tasks is likely to depend on the integrity of the hippocampus and temporal lobe (i.e. pattern separation, e.g., Yassa et al. 2010) and prefrontal cortex and anterior cingulate (i.e. working memory, Andrewes 2001; Lezak 1995). Normal performance on the tasks that contribute to attentional functions are likely to depend on integrity of subcortical brain regions including the basal ganglia as well as cortical regions such as the prefrontal and parietal cortices (Andrewes 2001; Lezak 1995). The presence of a relatively greater impairment in cognitive functions dependent on cortical and limbic brain regions (i.e., learning and working memory) with relatively subtle impairment in motor and attentional functions is consistent with neuropsychological models of AD which emphasise that cognitive impairment characteristic of both prodromal and clinically classified AD is disruption to memory and executive function (Baddeley et al. 1991; Kensinger et al. 2003; McKhann et al. 2011). This pattern of impairment is also consistent with the predilection of AD-related neuronal loss in the medial temporal lobe and other cortical brain areas (Jack et al. 2009; Villemagne et al. 2013).

Differences in the nature of impairment for the two cognitive composite scores were also evident in their sensitivity to detecting AD-related cognitive impairment in individuals. The learning/working memory composite was most sensitive to AD-related cognitive impairment with 100% of AD cases classified as impaired when the criterion for abnormality was set at a score of 90. When the criterion for abnormality was decreased to 80, the sensitivity for abnormality decreased to only 86% (Figure 1). As expected, the attention/psychomotor composite showed lower levels of sensitivity, with only 53% of AD cases identified when sensitivity was set at the least conservative level (i.e., score of 90). Taken together, these data indicate that with the use of these composite scores, cognitive impairment in AD will present as a relatively large impairment in working memory and learning and with relatively intact psychomotor and attentional functions. The nature and magnitude of this cognitive impairment is consistent with the descriptions of AD cases from the neuropsychological literature (Andrewes 2001; McKhann et al. 2011). While it is unsurprising that patients with clinical defined AD showed poor performance on a measure of learning and working memory, the high specificity of the learning/working memory composite, with the lesser impairment on the attention/psychomotor composite also indicates that the CogState tests themselves can be used effectively in patients with AD and suggests further that this pattern of performance may even be useful to clinicians investigating the aetiology of cognitive impairment in older adults.

As expected, in adults with MCI, cognitive impairment was qualitatively similar but quantitatively less pronounced to that observed for clinically diagnosed AD. Compared to healthy adults, the MCI group showed large impairment on the learning/working memory composite (*g* = 2.2), although not as great as that observed for the same composite in AD. While performance on the attention/psychomotor composite was also impaired compared to healthy adults, the magnitude of this impairment was only moderate (*g* = 0.51). Once again this impairment was less than that observed for the same composite in AD. Despite these impairment, performance on both the attention/psychomotor function and learning working memory composites in the MCI group was superior to that in the AD group. When considered for individuals, a score of ≤90 on the learning/working memory composite had optimal sensitivity and specificity for detecting cognitive impairment in MCI. At the optimum cut score for the attention/psychomotor composite, the sensitivity was only 40%, with a specificity of 85%. Therefore, as was observed for AD, cognitive impairment in MCI was characterised best as a large abnormality in working memory and learning with relatively normal psychomotor and attentional function. The likelihood that a combination of abnormal performance on the learning/working memory composite with normal performance on the attention/psychomotor composite could predict MCI or AD was very high, since individuals who met this criteria were 26 times more likely to have MCI or 30 times more likely to have AD than those who did not meet the criteria.

For the relationship between cognition and disease severity in the MCI and AD groups, while a significant linear relationship was observed between disease severity and the attention/psychomotor composite, this relationship was driven mainly by individuals with the most extreme scores on the severity measure. Furthermore the magnitude of this relationship was only small. In contrast to these more reflexive aspects of cognition, disease severity was strongly associated with the learning/working memory composite.

The second hypothesis that the attention/psychomotor composite and the learning/working memory composite would show high test-retest reliability and stability in healthy adults, adults with MCI and AD, was also supported. Assessments on the same tests conducted four times in three months showed that both composite scores remained stable and showed test-retest reliability with repeated administration. Thus, despite repeated testing over relatively short retest intervals, including in patients with cognitive impairment, both composites showed no evidence of practice effects, and estimates of within subject variability remained low. Further, estimates of test-retest reliability for each composite were, by convention, high (*r* > 0.70). These results are consistent with findings from earlier clinical studies of MCI and AD groups, which have shown that performance on the individual tests from the CBB show little to no practice effects, have high test-retest reliability, and have low within-subject variability (Darby et al. 2012; Fredrickson et al. 2010; Lim et al. 2013b). While individual measures from the CogState battery have been shown to be sensitive to cognitive decline in MCI (e.g. Lim et al. 2012a, b), it will be important now to determine the extent to which composite scores derived in this study will be also sensitive to cognitive decline in MCI.

Taken together, results of this study converge to suggest that the performance on the learning/working memory and attention/psychomotor composites of the CBB can be used to identify reliably cognitive impairment in people with, and at risk of AD. Thus the two composite scores from the CBB should be useful in screening for cognitive impairment in MCI or AD. The estimates of sensitivity for the composite scores from the CBB reported here are equivalent or slightly better than those reported previously for other screening instruments used commonly in the early identification of aMCI and AD. For example, estimates of the sensitivity for the MoCA show that the total score has a high sensitivity to AD, while retaining a high specificity. However, as was observed in the current study, the sensitivity of the MoCA to aMCI is also relatively high (81%; (Freitas et al. 2013)), provided that estimates of lower levels of specificity (e.g. 77%) are tolerated. As with the MoCA, performance on the MMSE also shows relatively high sensitivity and specificity for identifying cognitive impairment in AD (Freitas et al. 2013; Strauss et al. 2006) although its sensitivity to cognitive impairment in MCI is lower than the MoCA and that reported here, even if a low specificity is allowed. The equivalence of these estimates occurs mainly because all studies use the same method, where the test instrument is applied to identify cognitive impairment in a group of individuals that has been carefully assessed and undergone relatively rigorous inclusion and exclusion criteria. One strength of the composite scores, observed in this study, was that they were not associated with estimates of premorbid intelligence or depressive symptoms. The psychomotor attention composite was associated with levels of anxiety symptoms although the magnitude of this association was very small. Taken together this analysis of associations suggests that the composite cognitive scores may be useful in settings where issues such as low premorbid intelligence or mood obscure the assessment of cognitive function in individuals undergoing clinical workup for MCI or AD.

When cognitive assessments are conducted in unselected populations, such as in epidemiological studies, neuropsychological tests are always preferred to bedside screening instruments for the identification of cognitive impairment (Clarke et al. 2000; Ellis et al. 2009; Petersen et al. 2010). This is because neuropsychological tests provide more reliable estimates of individual cognitive functions. Acceptable estimates of validity and reliability are found for bedside screening instruments only when their total score is used, and accordingly, scores of their subscales have been shown to have limited use for describing the nature of cognitive impairment in individuals (Strauss et al. 2006). A limitation of bedside screening instruments for tracking cognitive function is reflected in their absence as outcome measures in clinical trials of drugs designed to improve cognitive function in MCI or AD. This is due to restriction in the range of possible scores for people with dementia; the presence of ceiling effects in data distributions; and the substantial practice effects that occur with repeated administrations. As with other neuropsychological tests, the tasks from the CBB have been used extensively in epidemiological studies, as well as in clinical trials (Bateman et al. 2011; Ellis et al. 2009). Furthermore associations between performance on the CBB tasks and that on conventional neuropsychological measures indicate that each task has sound construct validity (Maruff et al. 2009). The data shown here extend these findings to suggest that the two cognitive composite scores that arise from individual measures that comprise the CBB could be applied effectively as a cognitive screening instrument not only for assessing cognitive impairment in dementia, but also in other neurological and psychiatric conditions.

There are some limitations in the current study that warrant consideration in interpreting the results. First, as has been considered already the current data for this study were drawn from studies of MCI and AD, therefore the high sensitivity and specificity demonstrated here should be challenged in individuals from a clinical setting. Second, while the MCI group recruited here met clinical criteria shown to increase the risk of AD (Petersen et al. 1999), amyloid biomarkers (e.g., Petersen et al. 2010) were not measured in the current analysis. Therefore, although the current data show that the learning/working memory composite score was sensitive to the cognitive impairment that characterizes MCI more study is needed to determine the relationship the relationship between the CogState composite scores and amyloid biomarkers within this clinical classification. These issues notwithstanding the current results do show that the composite scores from the CogState Brief Battery have good potential for use in screening for cognitive impairment related to MCI and AD.
